# Evidence for higher order topology in Bi and Bi_0.92_Sb_0.08_

**DOI:** 10.1038/s41467-021-24683-8

**Published:** 2021-07-20

**Authors:** Leena Aggarwal, Penghao Zhu, Taylor L. Hughes, Vidya Madhavan

**Affiliations:** 1 Department of Physics and Materials Research Laboratory, University of Illinois Urbana-Champaign, Urbana, IL USA; 2 Department of Physics and Institute for Condensed Matter Theory, University of Illinois at Urbana-Champaign, Urbana, IL USA

**Keywords:** Surfaces, interfaces and thin films, Topological insulators

## Abstract

Higher order topological insulators (HOTIs) are a new class of topological materials which host protected states at the corners or hinges of a crystal. HOTIs provide an intriguing alternative platform for helical and chiral edge states and Majorana modes, but there are very few known materials in this class. Recent studies have proposed Bi as a potential HOTI, however, its topological classification is not yet well accepted. In this work, we show that the (110) facets of Bi and BiSb alloys can be used to unequivocally establish the topology of these systems. Bi and Bi_0.92_Sb_0.08_ (110) films were grown on silicon substrates using molecular beam epitaxy and studied by scanning tunneling spectroscopy. The surfaces manifest rectangular islands which show localized hinge states on three out of the four edges, consistent with the theory for the HOTI phase. This establishes Bi and Bi_0.92_Sb_0.08_ as HOTIs, and raises questions about the topological classification of the full family of Bi_*x*_Sb_1−*x*_ alloys.

## Abstract (web summary)

The experimental realization of higher order topological insulator (HOTI) in solid state materials remains elusive. Here, Aggarwal et al. reveal hinge states on three edges of both Bi and Bi_0.92_Sb_0.08_ (110) islands, supporting them as a class of HOTI.

## Introduction

HOTIs are topological crystalline insulators that have protected topological features on the boundaries with co-dimension greater than one. In contrast to (1st order), three-dimensional time-reversal protected topological insulators that show an insulating bulk with conducting surface modes, three-dimensional 2nd order HOTIs show insulating bulk and surfaces, but with one-dimensional conducting channels at hinges (i.e., the intersection of two surface facets) or equivalent crystal defects^1–6^. While several groups have recently succeeded in observing HOTI phases in an electronic circuit, phononic, and photonic systems^7–10^, the experimental realization and characterization of HOTIs in solid-state materials has proven more challenging. Among the proposed HOTI materials^4,11–16^, bulk Bi has recently emerged as a key candidate. Bi and Bi_1−*x*_Sb_*x*_ alloys are well-known important topological materials with highly tunable electronic properties. Scanning tunneling microscopy studies^1,17^ have observed signatures of one-dimensional modes on the edges of (111) facets of Bi, but the higher-order topological origin of these edge modes remains to be unequivocally established. Indeed an earlier study modeled the (111) facet as a free-standing Bi-bilayer^17^, which is predicted to be a quantum spin Hall insulator, and hence attributed the observed hinge modes to edge states of a quantum spin Hall system. In contrast, a recent study^1^ ascribed the metallic edge modes to the manifestation of a bulk HOTI protected by crystalline and time-reversal symmetries. New experimental data is necessary to distinguish between these scenarios. Importantly, the confirmation of Bi as a HOTI would indicate that the low-energy bands of bismuth must have trivial time-reversal protected ${{\mathbb{Z}}}_{2}$ topology^1,18^. This not only has implications for the topology of bulk Bi, which still remains controversial, but also the family of Bi_1−*x*_Sb_*x*_ alloys.

## Results

### STM measurements on Bi and Bi_0.92_Sb_0.08_ films

Bi naturally cleaves along the (111) plane, which makes STM studies of other planes, e.g., (110), difficult in bulk cleaved crystals. To circumvent this problem, we use molecular beam epitaxy (MBE) to grow Bi and Bi_0.92_Sb_0.08_ films on n-doped silicon (111) wafers^19–23^. The films were grown in a custom MBE system (described in more detail in the “Methods” section), and the composition was confirmed using Rutherford backscattering spectrometry (see Supplementary Note 4). Film thicknesses were chosen to be in the range of 30–90 bilayers (BLs) to ensure that they are representative of the bulk.

To establish a benchmark for comparison, we first reproduce the edge state data previously obtained on Bi(111) crystals using our films. STM topographies on our Bi(111)/Si(111) films reveal triangular islands and a hexagonal lattice indicative of (111) facets. The d*I*/d*V* spectra on the bulk and edges of the triangular islands show signatures of the Van Hove singularity associated with the surface states, as well as the emergence of clear 1D modes at type-A edges (see Supplementary Note 1), as was observed in the cleaved bulk Bi crystals^17^. Having confirmed the presence of edge modes in the (111) films, we move on to the (110) films.

**Fig. 1:**
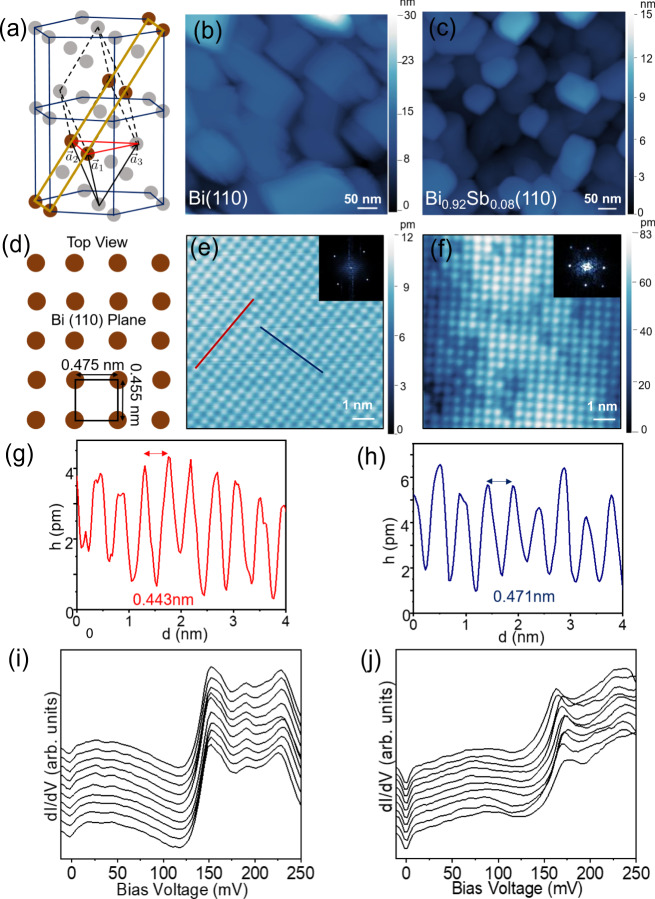
Atomic structure and STM measurements on Bi(110) and Bi_0.92_Sb_0.08_(110) films grown on silicon substrates. **a** Crystal structure of Bismuth with primitive cell, showing rhombohedral vectors (${\overrightarrow{a}}_{1}$,${\overrightarrow{a}}_{2}$,${\overrightarrow{a}}_{3}$). The (110) plane is indicated by a yellow rectangular box. (b,c) Large area (500 nm × 500 nm) topographic images of Bi(110) and Bi_0.92_Sb_0.08_(110) films respectively. The color bars show the relative height of the topography. **d** Top view of Bi(110) plane (as marked yellow rectangular box in (**a**)) with lattice constants. **e**, **f** STM images of 9 nm × 9 nm area on the rectangular facets of Bi(110) and Bi_0.92_Sb_0.08_(110) films (at 150 pA, 300 mV) respectively, with color bars indicating the relative height. Insets show the Fast Fourier Transform (FFT) topography (**g**, **h**) height (‘*h*’) profiles along the line cuts shown in (e) (marked in red and blue) showing the lattice periodicity of the pseudo-cubic crystal structure of Bi(110). **i**, **j** Representative d*I*/d*V* spectra near the centers of Bi(110) (ac modulation 3.5 mV and current 80 pA) and Bi_0.92_Sb_0.08_(110) (ac modulation 3.0 mV and current 120 pA) islands respectively. The spectra are vertically offset for clarity.

By changing the growth conditions (see “Methods” section), we were able to grow Bi and Bi_0.92_Sb_0.08_ in the (110) orientation on the same Si(111) substrates. STM images of the (110) oriented films of both Bi and Bi_0.92_Sb_0.08_ are shown in Fig. 1. Large area topographic images of bismuth films (Fig. 1b) show that the surface is uniformly covered with (110) oriented rectangular islands with longer edges parallel to the ($1\bar{1}0$) direction. Figure 1e shows an STM image with its corresponding Fourier transform in the inset. Height profiles along line cuts (red and blue color lines in Fig. 1e) confirm the lattice constants of the pseudo-cubic crystal structure of Bi(110), i.e., 0.443 and 0.471 nm in Fig. 1g, h, respectively. Representative spectra at the center of the Bi and Bi_0.92_Sb_0.08_ (110) islands, shown in Fig. 1i, j, reveal a broad increase in the density of states (DOS) above 130 mV in both samples. This increase can be ascribed to the spin-split Rashba surface states known to exist in bismuth and its alloys^19,24^.

### Spectroscopic measurements on a rectangular facet of Bi(110)

**Fig. 2:**
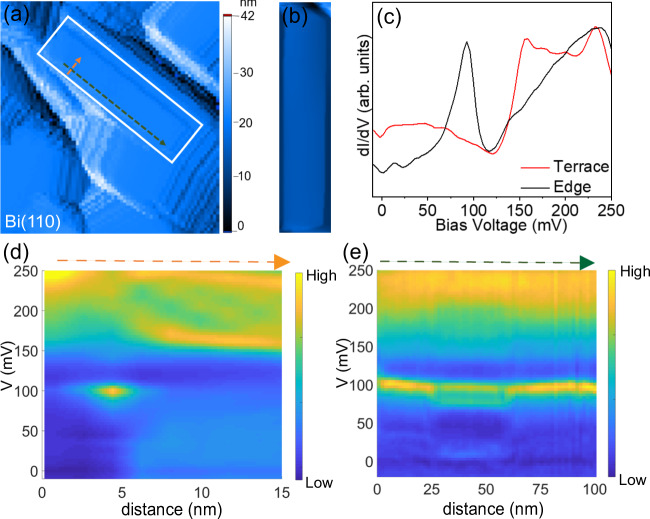
Spectroscopic measurements along and across the edge of a rectangular facet of a Bi(110) island. **a** Large area (250 nm × 350 nm) image of a Bi(110) film. The island of interest is marked by a white box. **b** Zoomed-in image of the Bi(110) island in (**a**). The color bar shows the relative height in the topography. **c** d*I*/d*V* spectra at the edge and center of the island (ac modulation 3.5 mV and current 100 pA). The edge spectrum shows a peak corresponding to the edge mode. **d**, **e** Intensity plot of STM spectra across (orange dashed line in (**a**)) and along (green dashed line in (**a**)) the step edge (ac modulation 3.5 mV and current 100 pA). The color bars indicate the relative height of the density of states.

**Fig. 3:**
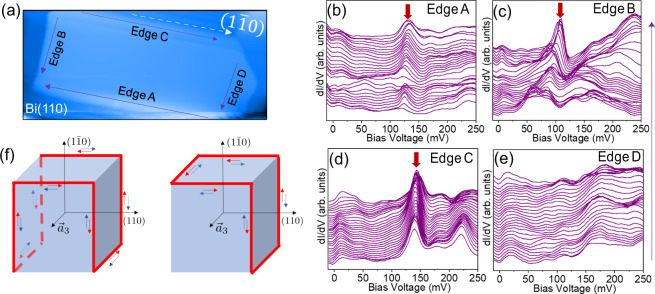
Metallic modes at the edges of the rectangular island of Bi(110). **a** Topographic image of a Bi(110) rectangular island of 168 nm × 80 nm area. The four edges of the island are marked as Edge A, Edge B, Edge C, and Edge D, respectively. The dotted white arrow shows the (1$\bar{1}$0) direction. **b**−**e** STM spectra (at ac modulation 3.5 mV and current 60 pA) along the four edges (as shown by purple arrows in (**a**)). A constant slope was subtracted from each spectrum for clarity. The raw data without slope subtraction is shown in Supplementary Fig. 3b, c, d, and e. The red block arrows indicate the sharp peaks corresponding to the edge modes that are only seen on three of the four edges. **f** Two possible configurations of helical hinge modes for Bi in the HOTI phase, where three edges of a rectangular island on a (110) facet hold helical modes. The ${\overrightarrow{a}}_{3}$ indicates a lattice vector of the Bi crystal as shown in Fig. 1a.

We first focus on the edge states observed on rectangular islands of the Bi (110) films. Spectra on the edge of the islands show a sharp peak in the DOS as shown in Fig. 2c. This singularity corresponds to bound modes which are localized within a few nanometers of the edge, as seen in the spectroscopic measurements across and along the edge shown in Fig. 2d, e respectively. This sharp peak resembles the DOS feature observed on the edges of Bi(111). To further characterize this edge mode, we obtain spectroscopic data on all four edges of a (110) oriented rectangular island as shown in Fig. 3b, c, d, and e. Intriguingly we find that only three out of four edges show clear edge modes, while the fourth edge shows no peaks. We confirmed this observation by measurements on different rectangular facet islands on multiple samples. In all cases, the edge that shows no edge modes is the one that is perpendicular to the ($1\bar{1}0$) direction. Crucially, the observation of edge states on three out of the four edges cannot be attributed to different bonding on just one side, which was a key part of the argument in ref. ^17^. By looking at the lattice structure of the (110) facet, we know that the two short sides are identical from a bonding perspective (see Supplementary Note 6). Yet, for a given island, one short side shows hinge states while the other does not.

### Theoretical justification

To understand the novel pattern of rectangular island edge modes on the (110) facets, let us first briefly review the recent developments for the topology of bismuth. Specifically, refs. ^1,18^ argued that the bulk topology of bismuth can be described as two copies of a strong 3D TI, i.e., two-band inversions at the *T*-point. Since there is an even number of strong TI copies, they conclude that the low-energy bulk bands of bismuth have trivial strong ${{\mathbb{Z}}}_{2}$ topology, but may support two robust Dirac points on high-symmetry surface facets exhibiting additional crystalline symmetries. For example, the (111) facet has ${\hat{C}}_{3}$ rotation symmetry; the two Dirac points are located at the *T*-point and belong to different ${\hat{C}}_{3}$ eigenvalue sectors having ${\hat{C}}_{3}$ eigenvalues −1 and $\exp (\pm {\mathrm{i}}\pi /3)$ respectively^1^. If the two Dirac points are pinned at the same energy, then they cannot be gapped out while preserving the ${\hat{C}}_{3}$ (no inter-cone gap) and time-reversal symmetries (no intra-cone gap). However, there are no symmetry constraints that fix the energies of the two cones, and generically it is expected that the (111) surface will be gapped^1^. In this context, ref. ^1^ considered the case where the (111) facet is the top facet of a hexagonal crystal (as shown in Fig. 1a). For this geometry, the ${\hat{C}}_{3}$ symmetry and time-reversal symmetry are compatible with sign-alternating surface-state masses/gaps on the side facets (this breaks ${\hat{C}}_{6}$ but preserves ${\hat{C}}_{3}$)^1^. Since the surface Dirac cones on the top/bottom (111) facets can also be generically gapped while preserving the symmetries, this will lead to sharply localized hinge modes where the side surfaces intersect the top/bottom surfaces, which were first observed for bulk Bi crystals in refs. ^1,17^, and confirmed for Bi films in our measurements above.

We propose that the observed edge states of an island on the (110) surface can be ascribed to ${\hat{C}}_{2}$ (of the bulk crystal around the $(1\bar{1}0)$ axis) and time-reversal symmetry protected higher-order topology consistent with ref. ^18^. The (110) surfaces of our islands do not have any protective crystal symmetry and thus we generically expect the two surface Dirac cones on those surfaces (as predicted by ref. ^18^) to be gapped. When considering hinge states though, we must also consider the mass gaps for surface states on the side-surfaces of such islands. First, the two long sides/edges of the (110) rectangular islands do not have any protected crystal symmetry, hence we expect the surface states to be gapped. Second, the two short sides/edges of the (110) rectangular islands are truncated ($1\bar{1}0$) surfaces, which could show remnants of the two gapless Dirac cone surface states. Indeed, ref. ^18^ recently emphasized that the ($1\bar{1}0$) surface has ${\hat{C}}_{2}$ rotation symmetry that protects the two, generically unpinned, surface Dirac points related by ${\hat{C}}_{2}$ rotation. In this case, the ($1\bar{1}0$) facet, cannot be gapped out (perturbatively) as long as the ${\hat{C}}_{2}$ and time-reversal symmetries are preserved since the Dirac cones do not coincide at a TRIM. However, since we are considering a surface island, translation symmetry is strongly broken on the ($1\bar{1}0$) facets. Hence, we do not expect the surface states to be robust since their momentum space locations become irrelevant. From these arguments, we expect that the surfaces that form the top and sides of the island can be generically gapped, so we can explore our observations in the context of higher-order topology in this system. In contrast to previous systems with crystalline-protected topology, (e.g., the mirror-protected surface Dirac cones in SnTe^25^), the (110) surface of Bi does not preserve the ${\hat{C}}_{2}$ bulk symmetry responsible for the higher-order protection^4^. However, this does not mean that we cannot observe signatures of higher-order topology, it just implies that the possible hinge state patterns may be affected by the details of the surface termination. For a rectangular crystal, as shown in Fig. 3f, where ($1\bar{1}0$) is the top edge/facet, there are two inequivalent configurations of surface mass gaps that match our observations of hinge modes on the (110) facet; i.e., that helical modes are localized on only three edges out of four. The configuration of helical hinge modes shown in the left panel of Fig. 3f nominally preserves both ${\hat{C}}_{2}$ (on the ($1\bar{1}0$) surface termination) and time-reversal symmetries, while the configuration shown in the right panel of Fig. 3f preserves only the time-reversal symmetry (details can be found in Supplementary Note 2). In addition, both configurations of helical hinge modes in Fig. 3f that are consistent with our observations, require broken inversion symmetry on the surface termination (which is a symmetry of the bulk of a bismuth crystal). However, this is not problematic since inversion is naturally broken for an island on a surface. Thus, we find that our observations corroborate, and are clearly indicative of higher-order topology in bismuth.

### Spectroscopic measurements on Bi_0.92_Sb_0.08_(110)

**Fig. 4:**
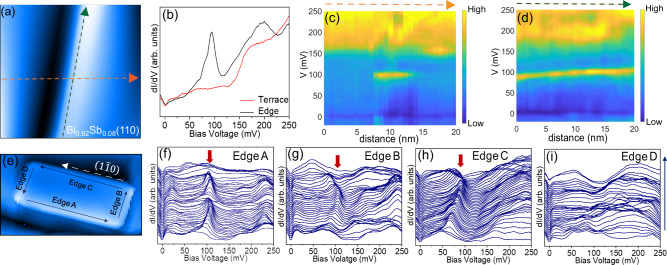
STM data on Bi_0.92_Sb_0.08_(110) film. **a** STM topographic image of 20 nm × 20 nm area of Bi_0.92_Sb_0.08_(110) film, showing an island edge. **b** d*I*/d*V* spectra at the edge and over the island terrace (at ac modulation 3.5 mV and current 100 pA), showing the peak around 100 meV corresponding to the mode at the edge. **c**, **d** d*I*/d*V* spectra obtained across (orange dashed line in (**a**)) and along (green dashed line in (**a**)) the island edge, respectively. The color bars indicate the relative height of the density of states. **e** Topography of rectangular facet of Bi_0.92_Sb_0.08_(110) with 50 nm × 25 nm area. The four edges are marked as Edge A, Edge B, Edge C, and Edge D, respectively. The white arrow along the edge shows the (1$\bar{1}$0) direction. **f**−**i** Spectroscopic data (at ac modulation 3 mV and current 120 pA) along the four edges A, B, C, and D respectively (following the blue arrows in (**e**)). A line with a constant slope was subtracted from all spectra for clarity. The raw data for these figures without slope subtraction is shown in Supplementary Fig. 3g−j. The red block arrows indicate the sharp peaks corresponding to the edge modes that are only seen on three of the four edges.

We can now use our established signature of higher-order topology in Bi to determine the topological nature of Bi alloyed with Sb. Previous theoretical studies have suggested that the Bi_1−*x*_Sb_*x*_ alloy undergoes a trivial-to-topological band-inversion transition at approximately *x* = 0.04^26–28^. However, similar to Bi, the narrow band gaps and the existence of multiple bulks and surface bands near the Fermi energy have made the theoretical and experimental determination of the topological properties of Bi_1−*x*_Sb_*x*_ complicated. From our studies on Bi, we propose that the pattern of the edge modes on the (110) oriented rectangular islands may provide a clear picture of the topology in this compound. Figure 4b shows STM spectra at an edge and plateau of the (110) facet of Bi_0.92_Sb_0.08_. A peak centered around 100 meV is observed at the edge, similar to that observed on Bi islands. Spectroscopic measurements across and along the edge shown in Fig. 4c, d respectively, reveal that this mode is localized to the edge. Importantly, similar to our data on Bi, we observe edge modes on three out of the four edges of the (110) oriented rectangular islands as shown in Fig. 4f−i. The explanation of this pattern of edge modes requires the system to be higher-order, and hence requires that the bulk has a trivial strong ${{\mathbb{Z}}}_{2}$ topology. Hence, our data on Bi_0.92_Sb_0.08_ suggests that it has a trivial strong ${{\mathbb{Z}}}_{2}$ topology and falls in the HOTI regime. This bears further study using other experimental techniques since it calls into question some long-accepted predictions about BiSb alloys, and indeed may have implications for the celebrated identification of nearby Bi_0.9_Sb_0.1_ as a strong ${{\mathbb{Z}}}_{2}$ topological insulator.

## Discussion

In summary, we have studied (110) oriented Bi and Bi_0.92_Sb_0.08_ films with STM and STS. We observe bound modes on three out of four edges of a rectangular island on (110) facets. Moreover, the edge showing no edge modes is always perpendicular to the ($1\bar{1}0$) direction. Theoretically, the novel pattern of the metallic edge modes observed on the (110) facets can be explained by a higher-order topology protected by time-reversal ($\hat{T}$) symmetry and bulk two-fold rotation (${\hat{C}}_{2}$) symmetry around the ($1\bar{1}0$)-axis^18^. Based on this we confirm that Bi and Bi_0.92_Sb_0.08_ belong to the HOTI class of topological materials. The latter result is particularly interesting since previous theoretical studies indicate that Bi_1−*x*_Sb_*x*_ with *x* ~ 0.08 should already be in a strong ${{\mathbb{Z}}}_{2}$ topological insulator phase heralded by a band inversion predicted at *x* ~ 0.04^26–28^. Our work brings new insights to the topology of Bi_0.92_Sb_0.08_ which is revealed as a new HOTI.

## Methods

### Sample preparation

Highly crystalline films of Bi and Bi_0.92_Sb_0.08_ were grown on n-doped silicon (111) wafers using a custom ultrahigh vacuum molecular beam epitaxy system. Before deposition, Si substrates were cleaned by ultrasonication in acetone and isopropanol. The substrates were then transferred to the MBE system (pressure < 2 × 10^−9^ Torr) and degassed at 520 ^∘^C for 10−12 h. After degassing, the substrate was cleaned by flash annealing, where they were repeatedly heated from 650 to 900 ^∘^C in 80 s. Each cycle helps to remove oxide layers or impurities from the silicon surface. The surface quality was confirmed by the observation of clear 7 × 7 reconstructed RHEED patterns. The substrate was rapidly cooled from 650 ^∘^C to room temperature at the rate of 1−2 ^∘^C/s after 16 cycles of flash annealing. For film growth, bismuth (purity ~ 99.9999%) and antimony (purity ~ 99.9999%) sources were evaporated from standard Knudcell cells at flux rates 0.0769 and 0.0071 Å/s respectively, as measured by a Quartz Crystal monitor. The substrates were held at room temperature during growth.

### STM measurements

Samples were transferred from MBE chamber to a vacuum suitcase at pressure < 3 × 10^−10^ and subsequently transferred to a Unisoku UHV-STM without exposure to air. All the measurements were done at 4.3 K. Normal tungsten tips were prepared by the etching method and used after cleaning by electron beam heating. All the topographic images were taken in constant-current mode and spectroscopic signals were recorded by standard lock-in amplifier with ac modulation 3−3.5 mV at 983 Hz frequency.

## Supplementary information

Supplementary Information

## Data Availability

The relevant datasets will be made available from the corresponding authors on reasonable request.
