# Supplementary material for: Evidence for higher order topology in Bi and Bi0.92Sb0.08
Source: Nat Commun. 2021 Jul 20;12:4420. doi: 10.1038/s41467-021-24683-8 (PMC8292335; doi:10.1038/s41467-021-24683-8)
Supplement: Supplementary file 1 — Supplementary Information [file 41467_2021_24683_MOESM1_ESM.pdf]

Supplementary information for
Evidence for Higher order topology in Bi and $Bi_{0.92}Sb_{0.08}$

Leena Aggarwal,[1] Penghao Zhu,[2] Taylor L. Hughes,[2] and Vidya Madhavan[1,*]

[1] *Department of Physics and Materials Research Laboratory, University of Illinois Urbana-Champaign, Urbana, Illinois 61801, USA*

[2] *Department of Physics and Institute for Condensed Matter Theory, University of Illinois at Urbana-Champaign, Illinois 61801, USA*

(Dated: June 29, 2021)

SUPPLEMENTARY NOTE 1: 1D HINGE MODES AT TYPE-A EDGES IN BI(111) FILM

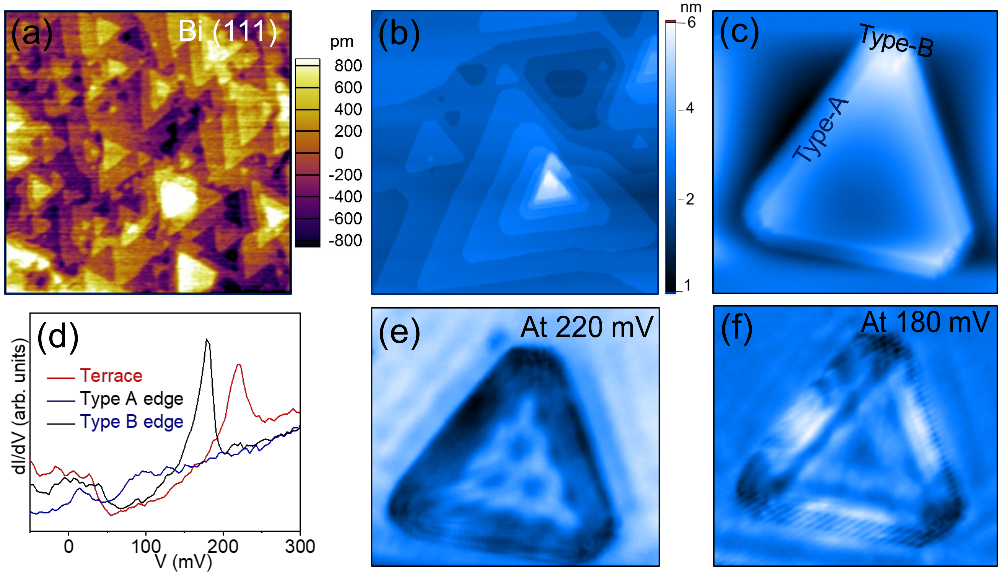

Supplementary Figure 1. **STM studies on Bi(111) films grown on silicon (111)substrates:** (a) A large area (700 nm ×700 nm) atomic force microscopy (AFM) image showing triangular islands which confirms the (111) orientation of the bismuth film with the color bar showing the relative height.(b) 300 nm ×300 nm STM image of the film with the color bar showing the relative height. (c) Topography of a 28 nm ×28 nm triangular (111) facet with two kind of edges, marked as Type-A and Type-B. (d) dI/dV spectra on the two types of edges and near the center of the island. The hinge mode density of states peak can be seen at the type-A edge. (e,f) STM dI/dV maps at 220 mV and 180 mV. The 180mV map clearly shows the localization of hinge modes at the three type-A edges.

SUPPLEMENTARY NOTE 2: THEORETICAL EXPLANATION FOR HELICAL EDGE MODES ON THE (110) FACET

Orientation of surface facets

As shown in Fig. 2 (a), in the Cartesian coordinate system we choose, the lattice vectors can be expressed as

$$\vec{a}_1 = (-\frac{1}{2}a, -\frac{\sqrt{3}}{6}a, \frac{1}{3}c), \quad \vec{a}_2 = (\frac{1}{2}a, -\frac{\sqrt{3}}{6}a, \frac{1}{3}c), \quad \vec{a}_3 = (0, \frac{\sqrt{3}}{3}a, \frac{1}{3}c), \tag{1}$$

where a and c are lattice constants as illustrated in Fig. 2 (a). As shown in Fig. 2 (b), the three corresponding reciprocal-lattice vectors defined by $\boldsymbol{b}_i \cdot \boldsymbol{a}_j = 2\pi\delta_{ij}$ with $i, j = 1, 2, 3$, are given by

$$\vec{b}_1 = g(-1, -\frac{\sqrt{3}}{3}, \frac{a}{c}), \quad \vec{b}_2 = g(1, -\frac{\sqrt{3}}{3}, \frac{a}{c}), \quad \vec{b}_3 = g(0, \frac{2\sqrt{3}}{3}, \frac{a}{c}), \tag{2}$$

where $g = 2\pi/a$. From Eq. (2), we can derive that

$$\begin{aligned} (111) &= \vec{b}_1 + \vec{b}_2 + \vec{b}_3 = g(0, 0, 3a/c), \\ (1\bar{1}0) &= \vec{b}_1 - \vec{b}_2 = g(-2, 0, 0), \\ (11\bar{2}) &= \vec{b}_1 + \vec{b}_2 - 2\vec{b}_3 = g(0, -2\sqrt{3}, 0), \\ (110) &= \vec{b}_1 + \vec{b}_2 = g(0, -2\sqrt{3}/3, 2a/c), \end{aligned} \tag{3}$$

from which we see that $(1\bar{1}0)$, $(11\bar{2})$, and (111) are the Cartesian x, y, and z directions (up to a sign), respectively. We can also see that (110), $(1\bar{1}0)$ and $\vec{a}_3$ are orthogonal to each other.

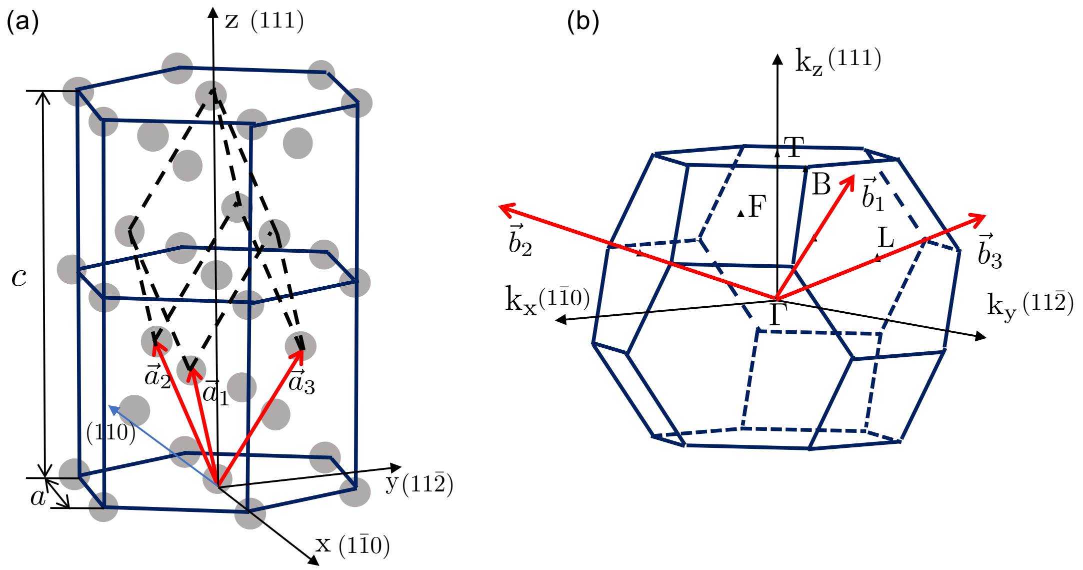

Supplementary Figure 2. (a) Crystal structure of Bi and/or BiSb alloys with primitive cell, indicating rhombohedral vectors ($\vec{a}_1$,$\vec{a}_2$,$\vec{a}_3$) and $(11\bar{2})$, (111), $(1\bar{1}0)$, (110) planes. (b) First Brillouin zone of Bi and/or BiSb alloys band structure.

Possible configurations of hinge modes under different symmetries

As discussed in the main text, the exotic three-sided hinge modes configuration observed on the rectangular island of a (110) surface facet is ascribed to higher order topology protected by the $\hat{C}_2$ symmetry around the $(1\bar{1}0)$-axis, and the time-reversal symmetry. We claim that it is necessary to break inversion symmetry to observe this hinge modes configuration. To support this claim, we enumerate all possible configurations of edge states when both $\hat{C}_2$ and inversion symmetries are preserved, only $\hat{C}_2$ is preserved, and only inversion is preserved. We also show two typical configurations which break both inversion and $\hat{C}_2$ rotation symmetries, one of which also gives the three-sided configurations. The enumeration shown in Fig. 3 clearly show that the three-sided edge modes configurations can only happen when the inversion symmetry is broken, irrespective of whether the $\hat{C}_2$ rotation symmetry is broken or not.

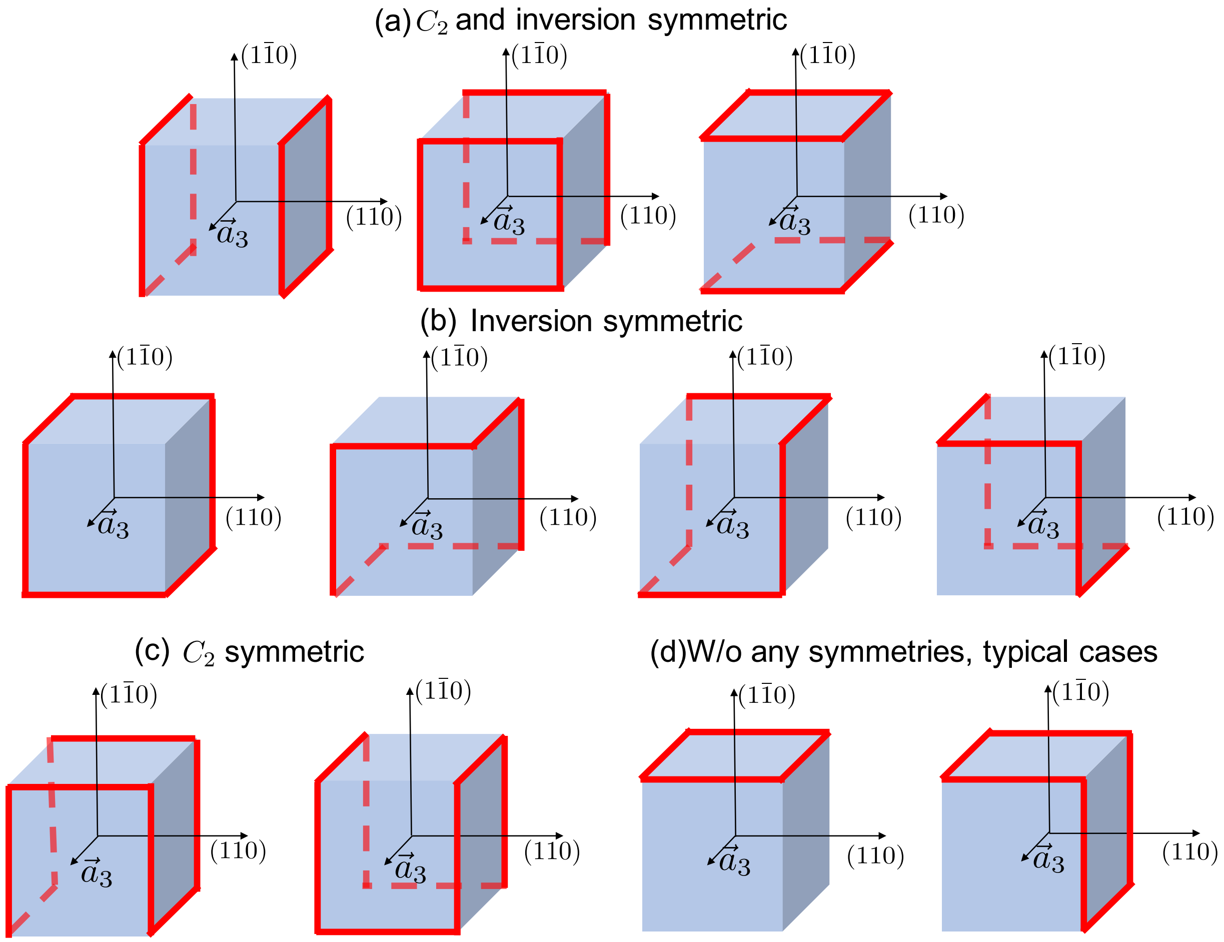

Supplementary Figure 3. All possible configurations of helical hinge modes on the (110) surface of Bi and/or $Bi_{0.92}Sb_{0.08}$ when (a) both $\hat{C}_2$ and inversion symmetries are preserved, (b) inversion is preserved but $\hat{C}_2$ is broken, and (c) $\hat{C}_2$ is preserved but inversion is broken. (d) shows two typical configurations when both $\hat{C}_2$ and inversion symmetries are broken. The red-color hinges are hinges where the surface mass vanish, *i.e.*, where there are gapless hinge modes. Note that $\hat{C}_2$ here refers to the two-fold rotation around $(1\bar{1}0)$ axis.

SUPPLEMENTARY NOTE 3: RAW DATA ON BI(110) AND BI$_{0.92}$SB$_{0.08}$(110) FILMS

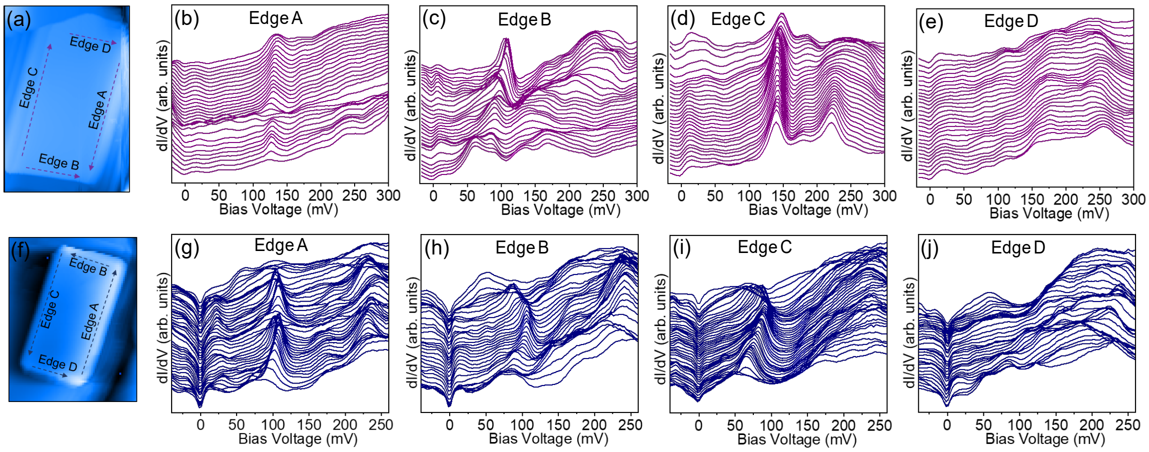

Supplementary Figure 4. (a) Topographic image of the of same Bi(110) rectangular island shown in Figure 3 (a) in the main manuscript. (b,c,d,e) Raw spectroscopic data (without slope subtraction) (at ac modulation 3.5 mV and current 60 pA) along the four edges marked Edge A , Edge B, Edge C and Edge D (indicated by purple arrows in Figure S4(a)). (f) Topographic image of the same Bi$_{0.92}$Sb$_{0.08}$(110) rectangular island as shown in Figure 4 (e) in the main manuscript. (g,h,i,j) Raw spectroscopic data (without slope subtraction) (at ac modulation 3 mV and current 120 pA) along the four edges marked Edge A, Edge B, Edge C and Edge D (shown as blue arrows along the edges in Figure S4(f)).

SUPPLEMENTARY NOTE 4: RUTHERFORD BACKSCATTERING SPECTROMETERY (RBS) TO CONFIRM SB COMPOSITION IN ALLOY

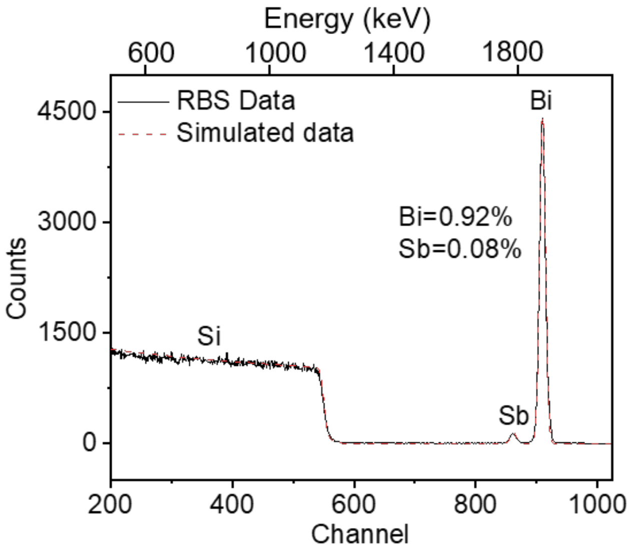

Supplementary Figure 5. RBS data (in solid black color) on BiSb alloy to confirm the composition of Sb with nicely fitted simulated data (in dashed red color line). Fitting confirms Sb composition 0.08%.

SUPPLEMENTARY NOTE 5: ADDITIONAL RAW DATA ON BI(110) AND $BI_{0.92}SB_{0.08}$(110) FILMS

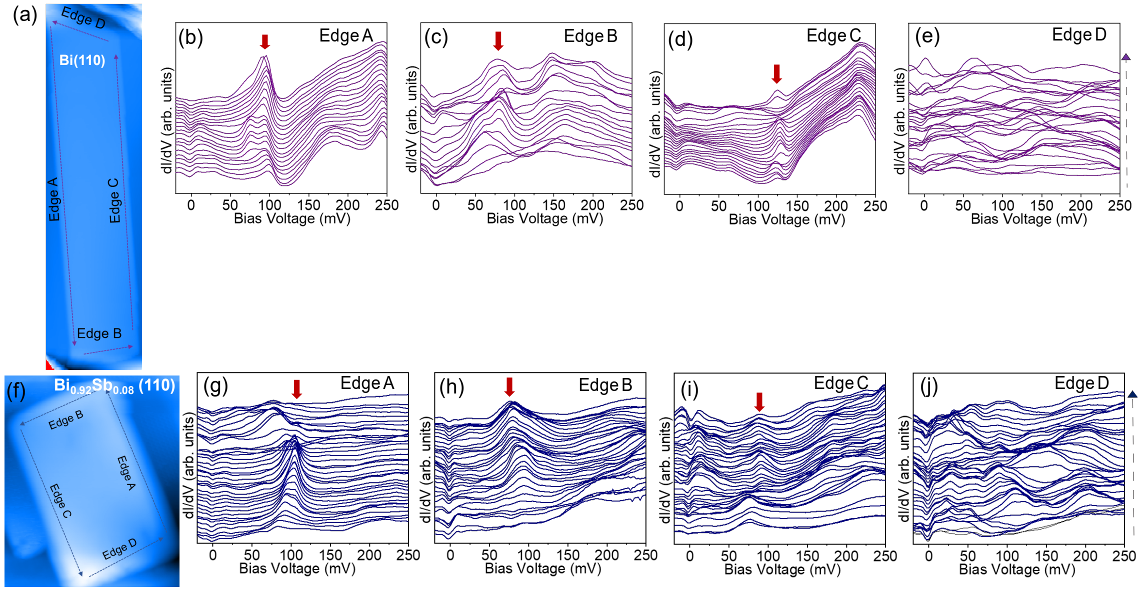

Supplementary Figure 6. (a)Topographic image of the of same Bi(110) rectangular island. (b,c,d,e) Raw spectroscopic data (without slope subtraction) (at ac modulation 3.5 mV and current 80 pA) along the four edges marked Edge A , Edge B, Edge C and Edge D (indicated by purple arrows in Figure S6(a)). (f) Topographic image of the same $Bi_{0.92}Sb_{0.08}$(110) rectangular island. (g,h,i,j) Raw spectroscopic data (without slope subtraction) (at ac modulation 2.5 mV and current 120 pA) along the four edges marked Edge A, Edge B, Edge C and Edge D (shown as blue arrows along the edges in Figure S6(f)). The red block arrows indicate the sharp peaks corresponding to the edge modes that are only seen on three of the four edges.

SUPPLEMENTARY NOTE 6: SCHEMATIC OF BI(110) LATTICE STRUCTURE

The schematic of Bi(110) lattice structure, as shown in Figure S7(a), aligned correctly with the long edge and short edge of (110) island. The atoms along the two long edges of the (110) island bond differently to the layer below. However, in our data, both long edges of the (110) islands always show edge states. Put another way, the two short edge sides are identical from a bonding perspective. But, for a given island, one short side shows hinge states while the other does not. Since the appearance hinge modes at three out of four edges has no connection with the geometry of the bonds at the edges, we can rule out bonding effects as an explanation for the pattern of our edge modes. Our data are instead well explained by the Higher order topological model.

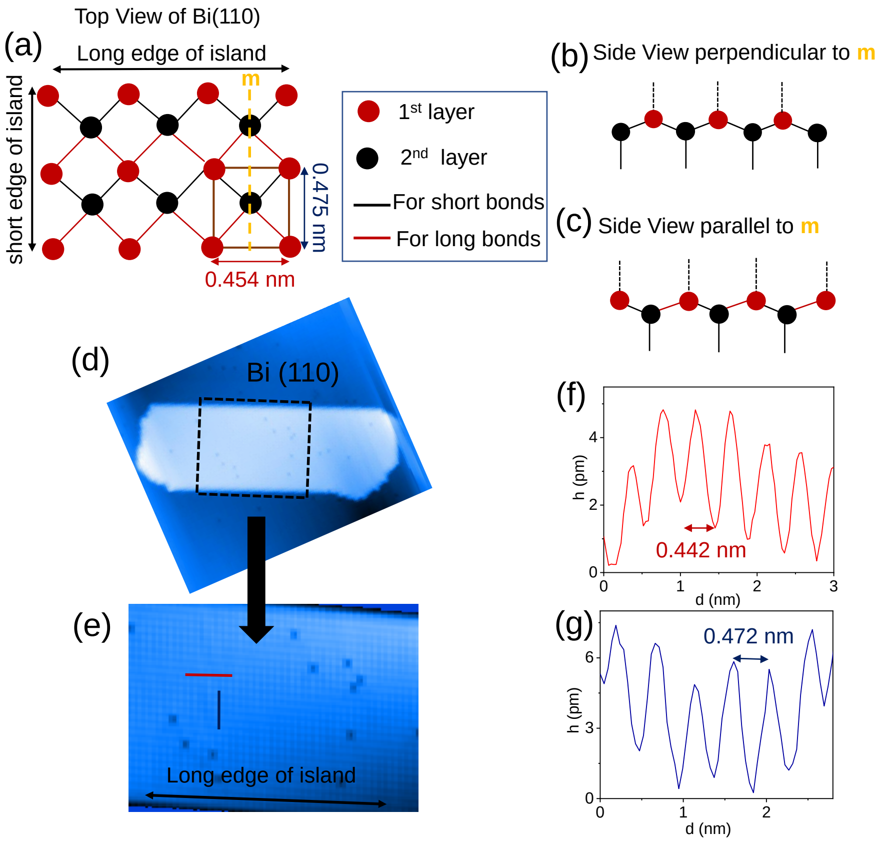

Supplementary Figure 7. (a) Schematic of the top view of first two layers of Bi(110) lattice structure, indicating two long edges of island bonded differently. Red atoms shows the first layer of two-dimensional rectangular lattice with lattice constants. The red lines and black lines between atoms represent long and short bonds respectively. The mirror plane ('m') of the structure are also shown as dashed yellow line.The lattice constants along long edge of island is 0.454 nm and short edge of island is 0.472 nm. (b, c) Side view of the lattice structure perpendicular and parallel to mirror plane ('m') respectively.(d) STM image of Bi(110) island shows the long and short edge sides of island same as in (a). (e) Zoomed image of Bi(110) island shown in (d), showing pseudo-cubic arrangements of atoms. (f,g) Lattice constants along the long edge side (corresponding red line in (e)) of island and short edge side (corresponding blue line in (e)) of the island respectively.

---

* To whom correspondence should be addressed: vm1@illinois.edu
